# Changes in cardiac volume determined with repeated enhanced 4DCT during chemoradiotherapy for esophageal cancer

**DOI:** 10.1186/s13014-018-1121-z

**Published:** 2018-09-18

**Authors:** Xue Wang, Jin-Zhi Wang, Jian-Bin Li, Ying-Jie Zhang, Feng-Xiang Li, Wei Wang, Yan-Luan Guo, Qian Shao, Min Xu, Xi-Jun Liu, Yue Wang

**Affiliations:** 1 School of Medicine and Life Sciences, University of Jinan-Shandong Academy of Medical Sciences, Jinan, Shandong Province China; 2 Department of Thoracic Radiation Oncology, Shandong Cancer Hospital affiliated to Shandong University, Shandong Academy of Medical Sciences, No. 440 Jiyan Road, Jinan, 250117 Shandong Province China; 3 Department of PET-CT Room, Shandong Cancer Hospital affiliated to Shandong University, Shandong Academy of Medical Sciences, Jinan, Shandong Province China; 4 Medical imaging department, Shandong Cancer Hospital affiliated to Shandong University, Shandong Academy of Medical Sciences, Jinan, Shandong Province China

**Keywords:** Esophageal cancer, Concurrent chemoradiotherapy, Heart volume change, Contrast enhanced four-dimensional computed tomography, Blood pressure

## Abstract

**Background:**

Concurrent chemoradiotherapy is considered curative intent treatment for patients with non-operative esophageal cancer. Radiation-induced heart damage receives much attention. We performed repeated four-dimensional computed tomography (4DCT) to detect changes in cardiac volume during radiotherapy for esophageal cancer patients, and explored potential factors responsible for those changes.

**Methods:**

Forty-six patients with esophageal cancer underwent enhanced 4DCT and three-dimensional (3D) CT scans before radiotherapy and every 10 fractions during treatment. The heart was contoured on 3DCT images, 4DCT end expiratory (EE) images and 4DCT maximum intensity projection (MIP) images by the same radiation oncologist. Heart volumes and other relative parameters were compared by the SPSS software package, version 19.0.

**Results:**

Compared with its initial value, heart volume was smaller at the 10th fraction (reduction = 3.27%, 4.45% and 4.52% on 3DCT, EE and MIP images, respectively, *p* < 0.05) and the 20th fraction (reduction = 6.05%, 5.64% and 4.51% on 3DCT, EE and MIP images, respectively, *p* < 0.05), but not at the 30th fraction. Systolic and diastolic blood pressures were reduced (by 16.95 ± 16.69 mmHg and 7.14 ± 11.64 mmHg, respectively, both *p* < 0.05) and the heart rate was elevated by 5.27 ± 6.25 beats/min (*p* < 0.05) after radiotherapy. None of the potential explanatory variables correlated with heart volume changes.

**Conclusions:**

Cardiac volume reduced significantly from an early treatment stage and maintained the reduction until the middle stage. The heart volume changes observed on 3DCT and 4DCT were consistent during radiotherapy. The changes in heart volume, blood pressure and heart rate may be valuable indicators of cardiac impairment and target dose changes.

## Background

Esophageal cancer ranked ninth for cancer incidence and sixth for cancer death in 2013, with 442,000 new cases and 440,000 deaths [1]. Concurrent chemoradiotherapy (CCRT) is considered to be a curative-intent treatment for patients with medically inoperable esophageal cancer [2]. Radiotherapy is an effective and relatively safe treatment for esophageal cancer [3], but can cause late locoregional complications such as esophageal, pulmonary and cardiac toxicity [4].

Concerns about radiation-related cardiac toxicity have been thoroughly discussed for patients with breast cancer and Hodgkin’s disease [5–11]. However, the maximum dose delivered to the heart is usually higher for patients with esophageal cancer than for patients with Hodgkin’s disease. Further, the irradiated volume of the heart is larger for esophageal cancer patients than for breast cancer patients [12].

Radiation-induced heart damage in esophageal cancer has received much attention. In a recent prospective analysis, Zhang et al. used single photon emission computed tomography to evaluate cardiac function in patients with esophageal cancer, and determined that cardiac impairment occurred during radiotherapy [3]. Using cone beam computed tomography (CBCT), Haj et al. prospectively investigated the changes in cardiac volume in esophageal cancer patients treated with neoadjuvant chemoradiation. The authors found that the cardiac volume was reduced in the early stage (41.4 Gy/1.8 Gy per fraction) of radiotherapy [13]. However, delineating soft tissue on CBCT is challenging, as the image quality is poorer than that of computed tomography (CT), and not all separate heart components are distinguishable by CBCT [14]. Four-dimensional computed tomography (4DCT) provides better image quality and can reduce artifacts caused by respiratory motion. Moreover, contrast enhancement in 4DCT is helpful for identifying the extent of tissues, which can reduce errors in organ delineation [15]. 4DCT is therefore considered to be a reliable and effective tool for assessing tumor and organ motion, as well as changes in tumor volume [16–21].

A reduction in heart volume suggests that subclinical cardiac damage may have occurred. Furthermore, changes in heart volume may influence the dose distribution of radiation to the tumor target and organs at risk. Consequently, changes in cardiac volume during radiotherapy for esophageal cancer require further exploration. In the present study, we explored the influence of radiotherapy on the heart volume by performing repeated 4DCT scans with intravenous contrast during the course of CCRT in patients with esophageal cancer. In addition, we assessed the correlations of age, gender, body mass index (BMI), disease staging, blood pressure, heart rate at baseline, blood pressure changes and heart rate changes with the cardiac volume reduction during CCRT.

## Methods

### Patients

Between September 2015 and January 2017, 58 patients with pathologically proven thoracic esophageal squamous cell carcinoma who were scheduled to receive CCRT in our department were consecutively enrolled in this study. None of the patients had a history of cardiovascular disease or chest radiotherapy. All patients had Karnofsky Performance Status values ≥80 and were considered physiologically fit for the therapy. This prospective study was approved by the Local Research and Ethics Committee at Shandong Cancer Hospital & Institute. Written informed consent was obtained from all patients prior to enrollment.

### CT simulation and image acquisition

All patients underwent contrast-enhanced three-dimensional CT (CE-3DCT) and CE-4DCT scans sequentially on a 16-slice CT scanner (Philips Brilliance Bores CT, Netherlands) while breathing. During the simulation, vacuum bags were used to immobilize patients in the supine position with their arms extended overhead. Three laser alignment lines were marked on the patients before CT acquisition. During radiotherapy, CE-4DCT and CE-3DCT images were acquired every 10 fractions with the patients in the same position.

During the 4DCT image acquisition, the respiratory signal was recorded with the Varian Real-time Positioning Management (RPM) gating system, which tracks the trajectory of infrared markers placed on the patient’s abdomen. The signal was sent to the scanner to label each CT image with a time tag. GE Advantage 4D software (GE Healthcare, Waukesha, WI, USA) sorts the reconstructed 4DCT images into 10 respiratory phases (labeled as 0–90%) on the basis of these tags, with 0% corresponding to end inspiration and 50% corresponding to 4DCT end expiratory (EE). Both the 3DCT and 4DCT images were reconstructed with a thickness of 3 mm, and the images were transferred to the Eclipse Treatment Planning System (Eclipse 8.6) for structure delineation and treatment plan generation. The 4DCT maximum intensity projection (MIP) was created from the 10 phases of 4DCT.

### Cardiac delineation and data acquisition

Heart contours were delineated according to the guidelines developed by Feng et al. [22], with the appropriate window settings (window width = 500 Hounsfield units, window level = 50 Hounsfield units). All delineations were performed by the same radiation oncologist to avoid inter-observer variation. For the determination of intra-observer variation, the heart volumes of six patients were delineated five times by the same radiation oncologist at one-week intervals. Together with the heart volume, the maximum heart distance (MHD) in the horizontal direction (reflecting the size of the heart contours) was measured automatically by the planning system.

### Blood pressure and heart rate measurement

During treatment, patients’ systolic blood pressure, diastolic blood pressure and heart rate were measured every day, according to international guidelines [23].

### Radiotherapy

The CE-3DCT and CE-4DCT scans before radiotherapy were registered based on bony landmarks by means of software tools in the Eclipse Treatment Planning System (Varian Medical Systems). The gross tumor volume was contoured on each phase of the 4DCT. The clinical target volume (CTV) was defined as the primary tumor, plus a 3-cm expansion superiorly and inferiorly along the length of the esophagus and a 0.5-cm radial expansion for each phase of 4DCT, then manually excluding the anatomical barriers (lung, trachea, large vessels, bones and heart) when not involved. The internal target volume was then constructed to envelop the CTV (10 CTVs in each phase of the 4DCT). The planning target volume was generated through the expansion of the internal target volume 0.5 cm in all directions.

All radiation treatments were delivered 5 days per week as either three-dimensional conformal radiotherapy or intensity-modulated radiotherapy, with 2.0 Gy per fraction and 6-MV photon beams. The patients were prescribed a total dose of 60 Gy. The main dose constraint was to limit the dose to both lungs to a V20 value (i.e., the percentage of the total lung volume receiving 20 Gy or more) of less than 30%. Other constraints included a V40 value < 50% for the whole heart and a maximum dose to the spinal cord of no more than 45 Gy.

### Chemotherapy

Patients in this study received one of two types of chemotherapy concurrently with their radiotherapy. Type A consisted of cisplatin (75 mg/m^2^ intravenously on day 1) and 5-fluorouracil (700 mg/m^2^ intravenous continuous infusion over 24 h daily on days 1–4) every 21 days. Type B consisted of capecitabine (625 mg/m^2^ twice a day by mouth on days 1–14) and oxaliplatin (85 mg/m^2^ intravenously on day 1) every 21 days.

### Calculation of radiation dose to the heart

The radiation dose to the heart was calculated from the heart volume receiving 20 Gy (V20), 30 Gy (V30), 40 Gy (V40), 45 Gy (V45) or 50 Gy or more (V50), as well as the average heart dose.

### Statistical analysis

Since the data were normally distributed, the results are presented as the mean ± standard deviation (mean ± SD). Changes in cardiac volume, MHD, blood pressure and heart rate during radiotherapy were analyzed with paired-sample t-tests. The associations between the change in heart volume and potential explanatory variables were examined by one-way analysis of variance or Pearson’s correlation analysis, as appropriate. The heart doses between fragments were analyzed by one-way analysis of variance. The initial heart volumes determined from 3DCT, EE and MIP images were compared through paired-sample t-tests.

Statistical analyses were performed with the SPSS software package, version 19.0, with *p* < 0.05 considered to be statistically significant.

## Results

### Patients

Patients with thoracic esophageal cancer were scheduled to undergo CE-3DCT and CE-4DCT scanning, with initial scans before radiotherapy and repeated scans at the tenth, twentieth and thirtieth fractions during radiotherapy. Those who failed to complete all the scans were excluded. Thus a total of 46 patients were included in this study. The patients’ characteristics are summarized in Table 1.

**Table 1:** Baseline characteristics and potential explanatory variables for cardiac volume reduction

Features	Value	*P*-value
Number of patients	46	
Age (years, Mean ± SD)	66.93 ± 9.76	0.281^a^
Gender (*n*, %)		0.107^b^
Female	14(30.43)	
Male	32(69.57)	
BMI (Mean ± SD)	22.63 ± 3.92	0.606^a^
Smoker (*n*, %)		0.410^b^
Yes	21(45.65)	
No	25(54.35)	
Hypertension (*n*, %)		0.309^b^
Yes	6 (13.04)	
No	40 (86.96)	
Tumor location (*n*)		
Upper	11	
Middle	22	
Lower	13	
Stage		0.137^b^
Stage II	13	
Stage III	33	
BP systolic at baseline (mmHg)	125.05 ± 15.98	0.881^a^
BP diastolic at baseline (mmHg)	75.66 ± 8.17	0.167^a^
HR at baseline (beats/min)	75.32 ± 6.23	0.412^a^
△BP systolic (Mean ± SD)	17.36 ± 17.96	0.160^a^
△BP diastolic (Mean ± SD)	4.32 ± 9.70	0.077^a^
△HR (Mean ± SD)	4.64 ± 4.91	0.448^a^

*Abbreviations*: *BMI* Body mass index, *BP* Blood pressure, *HR* Heart rate, *P*-value: the correlation between the potential explanatory variables and decline of heart volume. △BP systolic:systolic blood pressure at the twentieth – the initial systolic blood pressure. △BP diastolic: diastolic blood pressure at the twentieth – the initial diastolic blood pressure. △HR: heart rate at the twentieth – the initial heart rate

^a^Pearson’s correlation analysis

^b^ one-way ANOVA analysis

*P* ≥ 0.05 reflects that there was no correlation between the potential explanatory variables and heart volume change. Inversely,

*P* < 0.05 reflects that the potential explanatory variables were strongly linked to heart volume change

### Changes in heart volume and maximum heart distance during radiotherapy

Between the initial measurement (before radiotherapy) and the tenth fraction, the heart volume and MHD were markedly reduced at the middle location. On 3DCT, EE and MIP images, respectively, the heart volume was reduced by 3.27%, 4.45% and 4.52% (*p* = 0.027, *p* < 0.001 and *p* < 0.001), and the MHD was reduced by 0.98%, 1.40% and 1.29% (*p* = 0.035, *p* = 0.001 and *p* < 0.001). There were no significant changes in heart volume or MHD for the upper and lower locations at the tenth fraction. The results are presented in Table 2.

**Table 2:** Change of heart volume and MHD during radiotherapy (rate %, Mean ± SD)

Image	Location	0th–10th		0th–20th		0th–30th	
		HV (%)	MHD (%)	HV (%)	MHD (%)	HV (%)	MHD (%)
3DCT	upper	0.45 ± 4.35	−0.38 ± 2.09	3.06 ± 9.24	1.24 ± 3.29	−6.91 ± 15.34	−2.12 ± 4.81
	middle	3.27 ± 4.32*	0.98 ± 1.41*	6.05 ± 6.99*	1.82 ± 2.13*	2.64 ± 9.99	0.95 ± 3.50
	lower	1.06 ± 5.56	0.27 ± 2.00	−0.19 ± 10.79	−0.19 ± 3.56	2.44 ± 13.65	0.91 ± 4.78
EE	upper	−1.15 ± 4.51	−0.90 ± 1.42	1.10 ± 5.87	0.40 ± 2.28	−3.27 ± 12.11	−0.57 ± 4.38
	middle	4.45 ± 2.07*	1.40 ± 1.03*	5.64 ± 5.30*	1.88 ± 1.79*	2.83 ± 6.85	0.70 ± 2.68
	lower	0.10 ± 6.88	0.06 ± 2.27	1.14 ± 9.96	0.41 ± 3.16	2.72 ± 10.26	1.47 ± 4.03
MIP	upper	−1.54 ± 4.00	−1.24 ± 0.97	1.68 ± 6.17	0.20 ± 2.17	−3.12 ± 15.12	−0.71 ± 4.80
	middle	4.52 ± 2.40*	1.29 ± 0.84*	4.51 ± 5.02*	1.29 ± 1.50*	2.67 ± 7.26	0.62 ± 2.54
	lower	2.65 ± 9.12	0.28 ± 2.05	1.81 ± 9.05	0.52 ± 3.10	3.39 ± 10.96	1.19 ± 3.97

*Abbreviation*: *0th–10th* Comparison between the initial measure and the tenth fraction, *0th–20th* Comparison between the initial measure and the twentieth fraction, *0th–30th* Comparison between the initial measure and the thirtieth fraction, *HV* Heart volume, *MHD* Maximum heart distance, *EE* End expiratory of 4DCT, *MIP* Maximum intensity projection of 4DCT

*represent the *P* < 0.05

At the middle location, the heart volume was significantly lower at the twentieth fraction than at the initial measurement, with mean reductions of 6.05%, 5.64% and 4.51% on 3DCT, EE and MIP images, respectively (all *p* < 0.001). The MHD at the twentieth fraction for the middle location was reduced by 1.82%, 1.88% and 1.29% (*p* = 0.001, *p* < 0.001 and *p* < 0.001) on 3DCT, EE and MIP images, respectively. For the upper and lower locations, there were no significant changes in heart volume or MHD on the three types of images at the twentieth fraction.

The heart volume and MHD displayed a tendency to recover to the initial level at the thirtieth fraction. The differences in heart volume and MHD on the three types of images between the initial measurement and the thirtieth fraction were not significant (*p* > 0.05).

### Changes in blood pressure and heart rate during radiotherapy

**Fig. 1:**
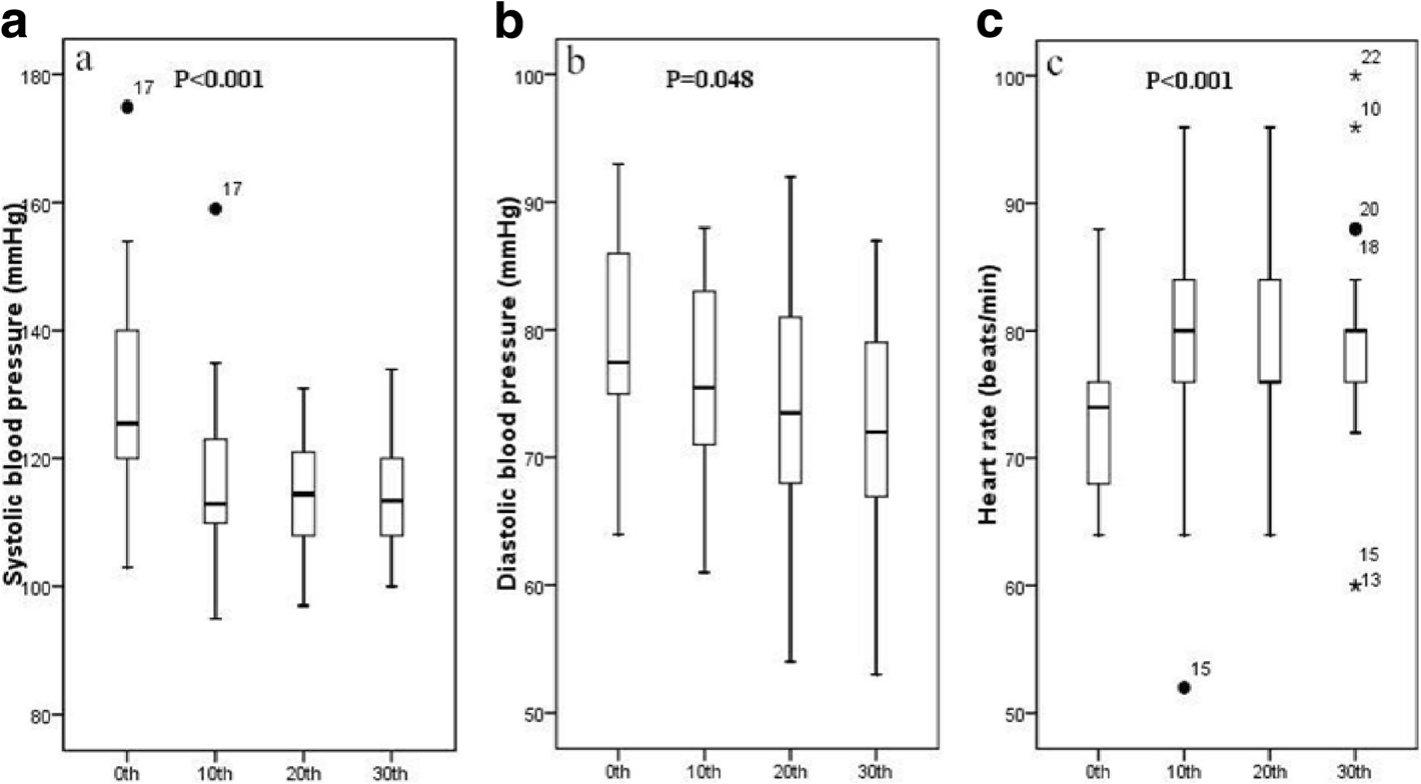
The changes of blood pressure and heart rate. This figure displays the changes of systolic blood pressure, diastolic blood pressure and heart rate during the course of treatment for the patients with mid-thoracic esophageal cancer. The abscissa represents the course of radiotherapy. The ordinate represents systolic blood pressure (**a**), diastolic blood pressure (**b**) or heart rate (**c**). Abbreviations: 0th = before radiotherapy; 10th = the tenth fraction of radiotherapy; 20th = the twentieth fraction of radiotherapy; 30th = the thirtieth fraction of radiotherapy (after radiotherapy)

Figure 1 displays the changes in systolic blood pressure, diastolic blood pressure and heart rate during the course of treatment for the patients with mid-thoracic esophageal cancer. Both systolic and diastolic blood pressure exhibited trends of decline during the entire treatment period at the middle location. At the tenth, twentieth and thirtieth fractions, respectively, systolic blood pressure was reduced by 15.64 ± 12.30 mmHg (*p* < 0.001), 17.36 ± 17.96 mmHg (*p* < 0.001) and 16.95 ± 16.69 mmHg (*p* < 0.001), and diastolic blood pressure was reduced by 2.91 ± 6.50 mmHg (*p* = 0.048), 4.32 ± 9.70 mmHg (*p* = 0.049) and 7.14 ± 11.64 mmHg (*p* = 0.009) from baseline.On the contrary, the heart rate at the middle location increased significantly from baseline, by 3.91 ± 7.92 (*p* = 0.031), 4.64 ± 4.91 (*p* < 0.001) and 5.27 ± 6.25 (*p* = 0.001) beats per minute at the tenth, twentieth and thirtieth fractions, respectively. However, at the upper and lower locations, no significant changes in blood pressure or heart rate were found during the entire treatment period.

### Comparison of cardiac doses among the three locations

As shown in Table 3, the heart dose was significantly greater for patients with mid-thoracic esophageal tumors than for patients with upper tumors (*p* < 0.05). Although the heart dose tended to be higher in the middle location than in the lower location, the difference between these two locations did not reach statistical difference (*p* > 0.05).

**Table 3:** Comparison of cardiac dose among different locations (rate %, Mean ± SD)

parameter	V20(%)	V30(%)	V40(%)	V45(%)	V50(%)	mean dose (Gy)
upper	17.24 ± 18.85	11.09 ± 12.35	5.15 ± 5.31	2.91 ± 3.15	1.48 ± 1.61	8.09 ± 7.58
middle	52.34 ± 22.13	39.48 ± 19.76	20.39 ± 11.07	12.99 ± 7.09	8.61 ± 5.79	2.49 ± 7.71
lower	41.80 ± 16.31	26.43 ± 9.44	16.73 ± 3.47	11.66 ± 1.17	8.40 ± 0.86	2.08 ± 6.47
*P*						
upper-middle	0.002	0.003	0.003	0.002	0.005	< 0.001
upper-lower	0.149	0.272	0.127	0.072	0.071	0.053
middle-lower	0.450	0.331	0.606	0.765	0.951	0.510

*Abbreviation*: *upper-middle* Comparison between the upper location between the middle location, *upper-lower* Comparison between the upper location and the lower location, *middle-lower* Comparison between the middle location and the lower location

### Factors potentially relevant to the decrease in heart volume

The age, gender, BMI, disease staging, systolic blood pressure at baseline, diastolic blood pressure at baseline, heart rate at baseline, decrease in blood pressure and increase in heart rate did not correlate significantly with the heart volume changes or the MHD (all *p* > 0.05). The data are summarized in Table 1.

### Comparison of heart volumes among the three types of images from the initial scans

Larger heart volumes (*p* < 0.05) were observed on the MIP images (mean value of 716.62 cm^3^) than on the 3DCT and EE images (680.96 cm^3^ and 685.02 cm^3^, respectively). The mean heart volumes on the 3DCT and EE images did not differ significantly from one another (*p* > 0.05). Consistent results were found for the MHD among the three types of images. The data are summarized in Table 4.

**Table 4:** Comparison of heart volume or MHD between the three images (cm^3^ or cm, Mean ± SD)

parameter	MIP-3DCT		MIP-EE		EE-3DCT	
	volume	MHD	volume	MHD	volume	MHD
3DCT	680.96 ± 133.75	10.88 ± 0.71	–	–	680.96 ± 133.75	10.88 ± 0.71
EE	–	–	685.02 ± 125.28	10.91 ± 0.66	685.02 ± 125.28	10.91 ± 0.66
MIP	716.62 ± 123.45	11.07 ± 0.64	716.62 ± 123.45	11.07 ± 0.64	–	–
Rate (%)	5.76 ± 4.41	1.71 ± 1.47	4.81 ± 2.45	1.47 ± 0.97	0.91 ± 3.77	0.24 ± 1.33
*P*	< 0.001	< 0.001	< 0.001	< 0.001	0.294	0.313

*Abbreviation*: *EE* End expiratory of 4DCT, *MIP* Maximum intensity projection of 4DCT, *MHD* Maximum heart distance, *MIP-3DCT* Comparison between MIP and 3DCT, *MIP-EE* Comparison between MIP and EE, *EE-3DCT* Comparison between EE and 3DCT

### Exclusion of intra-observer variation

The heart volumes of six patients were determined five times by the same radiation oncologist. No significant intra-observer variation was found (*p* > 0.05). The intra-observer error (5.40 ± 0.95 cm^3^) was significantly lower than the heart volume variation during radiotherapy (23.63 ± 14.11 cm^3^, *p* = 0.010).

## Discussion

The cardiac volume during radiotherapy was assessed by means of organ delineation through enhanced 4DCT, which can eliminate the effects of respiratory motion on the heart. In the early stages of CCRT, we found that the heart volume had decreased significantly from the initial measurement, with a decline of approximately 6%. Lutkenhaus et al. [14] used CBCT imaging retrospectively to investigate the changes in cardiac volume over the course of chemoradiotherapy for patients with esophageal cancer. Consistent with our study, those authors revealed that the cardiac volume was reduced with a median reduction of 8% between the initial measurement at week 1 and the measurement at week 4. However, they did not analyze the relationship between heart volume and tumor location.

The present study demonstrated that heart volume and MHD were reduced mainly in patients with tumors in the mid-thoracic region, rather than in the upper and lower locations. This was primarily due to the close proximity of the mid-esophagus to the heart. These results are also supported by the relative dose data: the V20, V30, V40, V45 and V50 values and the mean heart dose were greater when the esophageal tumor was in the middle region than in an upper or lower location. In a recent report, Zhang et al. observed new myocardial perfusion defects during radiotherapy in eight patients with esophageal cancer, and found that the defects were typically small fraction defects limited to the radiation field. They also discovered that the heart dose of patients with new myocardial perfusion defects were significantly larger than those of patients without new defects [3]. We therefore inferred that a high radiotherapy dose to the heart could be an important contributor to heart volume change. Ogino et al. concluded that the risk of symptomatic cardiac disease increased following high-dose and large-volume irradiation of the heart in long-term esophageal cancer survivors [12]. Based on above studies, precise radiotherapy technology and effective methods should be recommended to reduce the heart dose.

Although the reductions in heart volume and MHD in this study did not necessitate hospitalization or the use of cardiovascular drugs, some studies have suggested that radiotherapy damages the heart at both the cellular and molecular levels. For example, when the prescription dose for esophageal cancer accumulated to 40 Gy/20 fractions, a mild perfusion defect in the left ventricular anterior wall near the apex was found, which confirmed that the myocardium was damaged by radiotherapy [3]. During radiotherapy for esophageal cancer patients, troponin T levels increased significantly (by 0.007 units, *p* = 0.001) from baseline to week 5, providing an early sign of cardiac damage [13]. Additionally, in one study [24], ionizing radiation was reported to cause non-transient alterations in the cardiac mitochondria, which ultimately may have caused heart muscle malfunction. At a microscopic level, not only did collagen levels increase as a whole, but the proportion of type I collagen increased relative to that of type III collagen. This change was thought to alter the compliance of the myocardium, thus contributing to diastolic dysfunction [25].

Diastolic dysfunction is now recognized as a common cause of heart failure and poor outcomes, even when the left ventricular ejection fraction is preserved [26, 27]. Thus, we assumed that reduced cardiac diastolic function could partly explain the reduced heart volume in the present study. Diastolic dysfunction was observed in patients who received mediastinal radiation of > 35 Gy for Hodgkin’s disease [28]. Hatakenaka et al. concluded that direct radiation to the left ventricle predominantly impaired diastolic function by restricting wall motion in the radiated areas [2]. Consistently, Zhang et al. found that wall motion and wall thickening decreased significantly during radiotherapy in patients with esophageal cancer [3].

In the present study, we also assessed potential parameters that could predict the observed decline in heart volume. However, we found no correlations between the cardiac volume reduction and the patients’ baseline characteristics. On the contrary, Haj et al. found that the decrease in heart volume correlated significantly with the systolic blood pressure at baseline, diastolic blood pressure at baseline and heart rate at baseline in patients with esophageal cancer [13]. The reasons for these differing results are unclear. Larger sample sizes and extensive multivariate analyses will be essential in further research.

The patients in our study had squamous cell carcinomas and received total radiation doses of up to 60 Gy in CCRT, much higher than the neoadjuvant chemoradiation dose for esophageal cancer (approximately 40 Gy) and the CCRT dose for adenocarcinoma (approximately 50.4 Gy). It should be noted that a change in heart volume immediately after CCRT (60 Gy) has not been reported. Our results indicated that the heart volume and MHD tended to recover to the initial level at the thirtieth fraction. Edmunds et al. found that the cardiac volume had increased by 1.7% (*p* = 0.747) 3 months after radical radiotherapy for esophageal cancer [29]. The reasons for this phenomenon are poorly understood, but we have considered several possibilities. Firstly, at the thirtieth fraction, the cardiomyocyte edema became serious and the cardiac repair became more obvious than the injury. Secondly, dehydration was reported to be the main reason for hospital admission during CCRT for esophageal cancer [30]; therefore, the recovery of the depleted intravascular volume in the later stages of CCRT may have increased the heart volume. Moreover, in our study, the hearts were outlined along the pericardium, so changes in the pericardium may have been responsible for the observed results. Martel et al. [31] studied the effects of the radiation dose and volume on the pericardium in 57 patients with localized esophageal carcinoma. Nonmalignant pericardial effusions occurred in five patients, and the effusions were found at least 3 months post-radiation treatment. However, we did not observe significant pericardial effusion at the end of radiotherapy. Therefore, the mechanisms responsible for the change in heart volume after radiotherapy require further study.

While the heart rate increased during radiotherapy in the present study, both systolic and diastolic blood pressure decreased significantly. Similarly, Haj et al. discovered that systolic and diastolic blood pressure decreased and the heart rate increased in patients treated with neoadjuvant chemoradiation [13]. In Hatakenaka’s [2] study, the LV-STVI and heart rate exhibited opposite trends, but the cardiac output index did not change. The authors reasoned that the cardiac output index remained the same because the elevated heart rate had compensated for the reduced LV-STVI. Zhang et al. explained that the acceleration of the heart rate may have been due to radiotherapy-induced damage to the heart conduction system [3]. And many reports concluded that conduction abnormalities such as sick sinus syndrome, ventricular arrhythmias, bundle branch blocks and complete heart blocks can be caused by RT [32–34].

To study the changes in cardiac volume during radiotherapy, we used 4DCT, which explicitly includes temporal changes in anatomy during the imaging process [17], and thus can provide spatial and temporal information on the heart during respiration. Moreover, CE-4DCT is a feasible technology that has been applied in the clinic [35]. The coefficients of variation for the gross tumor volumes delineated on CE-4DCT images are smaller than those delineated on plain scan images, indicating that CE-4DCT can reduce the error of organ delineation [15]. Our results demonstrated that the initial heart volumes determined from MIP images were larger than those determined from 3DCT and EE images, while the heart volumes determined from 3DCT and EE images did not differ significantly from one another. The trends in heart volume changes observed on these three types of images were consistent during radiotherapy. Thus, the volume changes were not due to differences among the various image types, further confirming that the cardiac volume decreased in the early stages of radiotherapy.

Morphological changes resulting from heart volume variation could result in inaccurate dose delivery to the tumor and organs at risk [14]. Since volume changes mainly occurred around the twentieth fraction of radiotherapy, modification of the treatment plan at this time point would probably be beneficial for suppressing the tumor and protecting the heart from radiotherapy.

The patients in the present study received chemotherapy at the same time as radiotherapy. Chemotherapeutic drugs are known to have cardiotoxic effects; for instance, they may cause arrhythmias [36–41]. However, there has been little research on cardiac volume reduction resulting from chemotherapy. Thus, the possible effects of chemotherapy on the cardiac volume should be studied further to determine the generalizability of our findings.

Our study may have been impacted by some limitations. Blood samples were not collected, so we did not use biomarkers to research the function of the heart. In addition, because long-term follow up is not carried out after radiotherapy, the long-term effects of radiotherapy on the cardiac volume could not be determined.

## Conclusions

We discovered that the heart volume and MHD decreased significantly in the early stages of CCRT for esophageal cancer, but we found no symptomatic heart disease during treatment. The blood pressure decreased, while the heart rate increased significantly during the course of treatment. The cardiac dose was higher for the middle location than for the upper and lower locations. The observed changes in cardiac volume may have been caused by the relatively high dose of radiation. These changes may be valuable indicators of cardiac impairment necessitating target dose changes.

## Abbreviations

3DCT: Three-dimensional computed tomography
4DCT: Four-dimensional computed tomography
BMI: Body mass index
CBCT: Cone beam computed tomography
CCRT: Concurrent chemoradiotherapy
CE: Contrast-enhanced
CTV: Clinical target volume
EE: End expiratory
MHD: Maximum heart distance
MIP: Maximum intensity projection
RPM: Real-time Positioning Management
